# Analysis of Al_2_O_3_ Nanostructure Using Scanning Microscopy

**DOI:** 10.1155/2018/8459768

**Published:** 2018-05-14

**Authors:** Marek Kubica, Władysław Skoneczny, Marek Bara

**Affiliations:** Faculty of Computer Science and Materials Science, University of Silesia, Bankowa 12, Katowice, Poland

## Abstract

It has been reported that the size and shape of the pores depend on the structure of the base metal, the type of electrolyte, and the conditions of the anodizing process. The paper presents thin Al_2_O_3_ oxide layer formed under hard anodizing conditions on a plate made of EN AW-5251 aluminum alloy. The oxidation of the ceramic layer was carried out for 40–80 minutes in a three-component SAS electrolyte (aqueous solution of acids: sulphuric 33 ml/l, adipic 67 g/l, and oxalic 30 g/l) at a temperature of 293–313 K, and the current density was 200–400 A/m^2^. Presented images were taken by a scanning microscope. A computer analysis of the binary images of layers showed different shapes of pores. The structure of ceramic Al_2_O_3_ layers is one of the main factors determining mechanical properties. The resistance to wear of specimen-oxide coating layer depends on porosity, morphology, and roughness of the ceramic layer surface. A 3D oxide coating model, based on the computer analysis of images from a scanning electron microscope (Philips XL 30 ESEM/EDAX), was proposed.

## 1. Introduction

Nanotechnology is a new research approach, which refers to the understanding and improvement of the properties of matter on the nanoscale. In such a dimension, matter shows completely different and often surprising properties, as a result of which the traditional boundaries between scientific and technical disciplines become blurred. Anodic oxide conversion coatings obtained in the electrodeposition process are the components of a wide variety of nanostructures, such as photonic crystals, metamaterials, and microelements. They can also be used as matrices for the production of nanotubes or nanowires [1–4].

**Figure 1:**
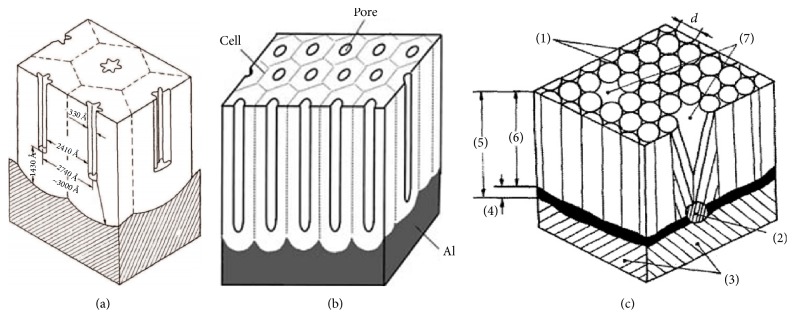
Al_2_O_3_ oxide layer models by (a) KHR, (b) Sulka, and (c) Skoneczny.

A number of theories presented in the literature, which deal with the structure, formation mechanisms, and the forming of Al_2_O_3_ coatings, result from the analysis of microscopic images and physicochemical properties of the coatings. The first author of Al_2_O_3_ coating formation theory was Csokan [5]. It says that, in the beginning of the anodizing process, oxygen atoms or electrolyte anions are adsorbed or chemisorbed onto active sites (defects, faults, and grain boundaries) on the aluminum surface. Then the layer of oxide nuclei is formed. Perpendicular oxide growth is much slower than at the edges of the nucleus, and as a result of this lateral growth, the oxide covers the entire aluminum surface. The local differences in chemical solubility of the oxide layer and in structural deformations created by different state of energy are directly responsible for the formation of pores. Keller, Hunter, and Robinson (KHR), the authors of [6], extended Csokan's theory. In their opinion at the beginning of the hard anodizing process, the highly ordered homogeneous anodic oxide barrier is created. The further current applied causes the rise of the porous oxide layer. The passing current increases the local temperature of the electrolyte such that consequently oxide dissolution is enhanced and current breakdowns form pores in the oxide layer. The anodic structure exhibiting the hexagonal arrangement of cells is derived from pores due to the existing tendency of spherical distribution of potential and current about the pore. The barrier layer thickness and pore and cell dimensions depend on the voltage applied in the process of hard anodizing, type of electrolyte, its composition, and process temperature. KHR coating was formed at 120 volts in the 4% phosphoric acid electrolyte and temperature of 297,15 K (Figure 1(a)) [6].

Another author of oxide layer structure formation theory was Sulka [7]. He represented schematically oxide layer created by hard anodizing as a closed-packed array of hexagonally arranged cells containing pores in each cell center (Figure 1(b)). In his opinion, growth of the oxide layer takes place at the metal and oxide interface at the pore bottoms and involves the conversion of a preexisting, naturally occurring film on the surface into the barrier-type film and further into a porous oxide layer. During the porous oxide growth, a thin and compact barrier layer at the pore interface is continuously dissolved by locally increased field, and a new barrier layer at the interface is rebuilt. Steady-state growth causes the cylindrical pores to appear. Nanostructures were produced by 1–3 steps of anodizing in sulphuric acid at electrolyte potentials between 15 and 25 V at temperature of 274.15–283.15 K [11].

The oxide coating layer model proposed by Skoneczny is shown in Figure 1(c) with marks: (1) micropores, (2) admixture, (3) metal, (4) barrier layer, (5) oxide film, (6) porous layer, (7) macropores, and (d) fiber diameter. The model obtained in a three-component SAS electrolyte (aqueous solution of acids: sulphuric 33 ml/l, adipic 67 g/l, and oxalic 30 g/l) at temperature of 293 K and current density of 200 A/m^2^ is comprised of a thin barrier layer directly adjoining metal and a porous upper layer. Micropores formed as a result of aluminum oxide nanofibers contacting one another are the result of the formation of the columnar structure. A section of a pore has an equilateral triangle shape, where the sides have been replaced by an arc formed from a circular section. Energy interferences in the oxide layer and the local etching of grain boundaries of the substrate material and mixtures caused the formation of macropores [12]. The research discussed was based on a model proposed by Skoneczny due to the fact that it had been produced under similar conditions to those that were used in the experiments.

**Figure 2:**
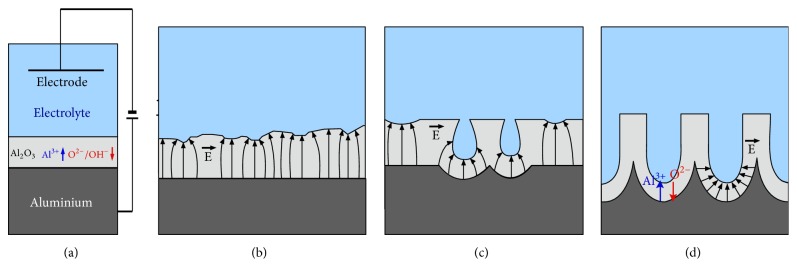
Phases of forming the Al_2_O_3_ layer by means of hard anodizing: (a) the formation of a barrier layer, (b) local field distribution caused by surface fluctuations in the surface, (c) the formation of pores through layer's dissolution, and (d) stable growth of the layer.

Anodic coatings produced on aluminum are applied in tribology to increase the hardness, as well as to improve wear resistance and corrosion resistance. Physicochemical characteristics of anodic oxide coatings (AOC) are determined on the basis of the examination of their structure and morphology, which have a decisive influence on the tribological properties. The latter, in lubricant-free polymer/layer systems, depend mainly on the porosity, morphology, and roughness of the oxide layer's surface and the substrate material used. The type of the electrolyte used has a fundamental influence on the appearance and, consequently, the tribological properties of the oxide layer. Different acids or their mixtures can form the electrolyte. Parameters such as current density, anodizing time, temperature of electrolysis, electrolyte stirring rate, and the type of base material are of importance. Controlling them allows us to obtain coatings with predefined properties [13]. The phases of forming the Al_2_O_3_ (Figure 2) layer can be divided into the following: the formation of a barrier layer, local field distribution caused by surface fluctuations in the surface, the formation of pores through layer's dissolution, and stable growth of the layer [14, 15].

**Figure 3:**
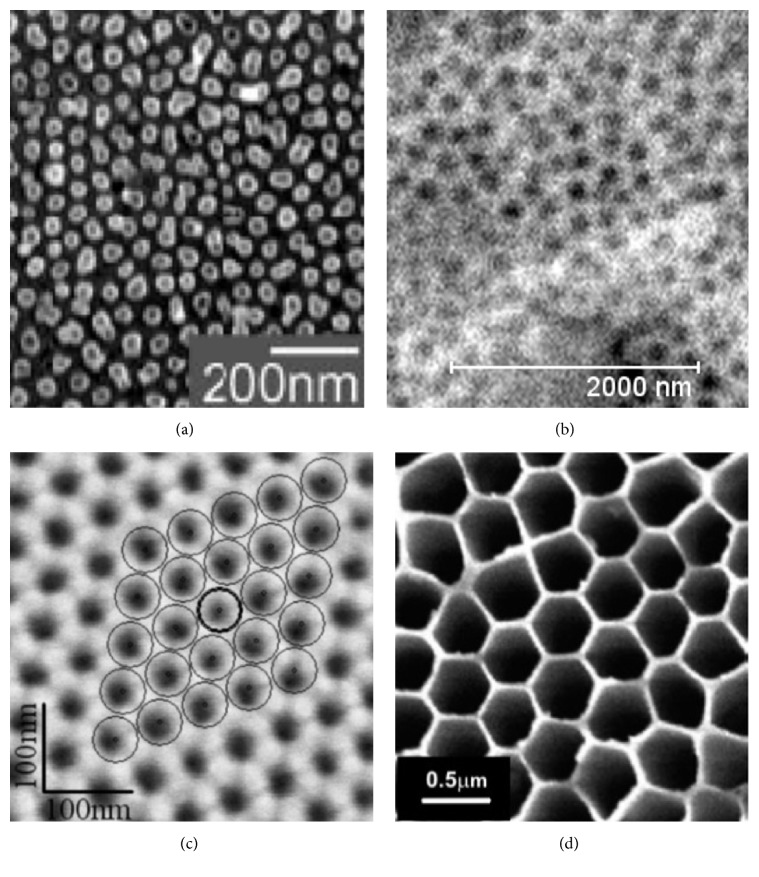
SEM bottom view of a porous alumina layer prepared in different conditions: (a) H_3_PO_4_, 160 V, 276.15 K [7], (b) mixture of (CH_2_)_4_(COOH)_2_, H_2_SO_4_, and H_2_C_2_O_4_, 300 A/m^2^, 303.15 K [8], (c) 2.4 M H_2_SO_4_, 25 V, 281.15 K [9], and (d) H_2_SO_4_ and H_3_PO_4_, 120 V, 308.15 K [10].

Analysis of images from a scanning microscope presented in the literature has shown that multiple structures of the oxide layers' surface morphology can be obtained, depending on the applied conditions of their formation through anodizing (Figures 3(a)–3(d)).

Based on a computer analysis of the microscopic images obtained, an attempt was made to reproduce the real surface morphology of the Al_2_O_3_ layer in a program (SFO 2012) [16]. The idea of the developed SFO 2012 program is to make it possible to simulate the morphology of the oxide layer, Al_2_O_3_, produced in different hard anodizing conditions.

## 2. Layer Technology

The EN AW-5251 aluminum alloy was the base for Al_2_O_3_ layer. The chemical composition of EN AW-5251 is as follows: Mg, 1.9%; Mn, 0.26%; Si, 0.2%; Fe, 0.32%; Cu, 0.05%; Zn, 0.01%; Cr, 0.02%; Ti, 0.02%; and Al, 97.2%. It was chosen because of its good mechanical properties and a scanty content of admixtures of other elements, which facilitated the formation of the oxide layer. Coatings were produced in the anodizing system on rolled sheet metal strips of an area of 1 × 10^−3^ m^2^ and thickness of 4 × 10^−3^ m.

Prior to the oxidation process, the surfaces of the samples were etched in a 5% solution of KOH and next, in order to reverse the etching reaction, tin-plated in a 10% HNO_3_ solution. Distilled water was used for rinsing after the etching and tin-plating procedures. Oxidation of the properly prepared sample surfaces was carried out in a SAS solution of the following acids: sulfuric 33 ml/l, adipic 67 g/l, and oxalic 30 g/l. Temperature of the electrolyte was 293–313 K, current density was 200–400 A/m^2^, and oxidation time was 40–80 minutes. The electrolyte was stirred at a constant rate of 150 rpm. The production parameters of the individual samples are presented in Table 1. The range of anodization parameters was determined for three levels: minimal, central, and maximal. The minimum and maximum parameters correspond to the boundary values normally used in the production of Al_2_O_3_ layers.

**Figure 4:**
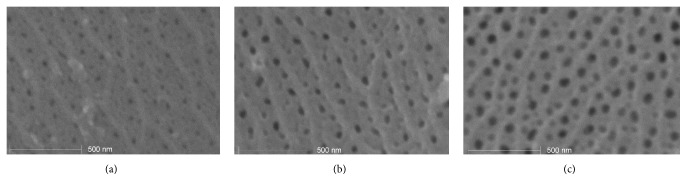
Morphology of the oxide layer: (a) sample A, (b) sample B, and (c) sample C.

 The anodizing samples with a deposited oxide layer (Figure 4) were rinsed in distilled water.

## 3. Results and Discussion

### 3.1. Analysis of the Chemical Composition of the Substrate and Al_2_O_3_ Layer

**Figure 5:**
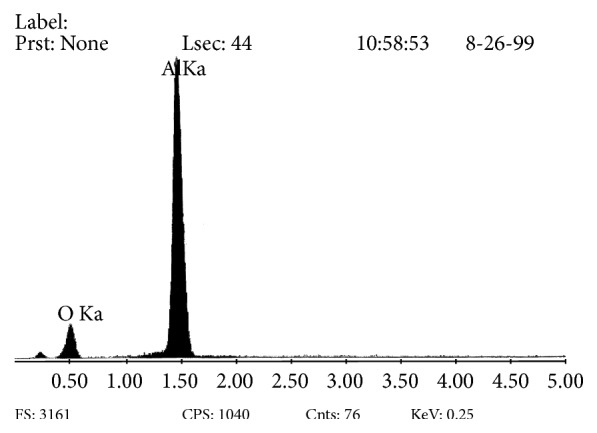
Results of the chemical composition analysis of the substrate and Al_2_O_3_ layer.

Analysis of the chemical composition was performed on a transverse microsection of the Al_2_O_3_ layer produced on an aluminum alloy in a three-component electrolyte in order to corroborate and examine the amounts of elements of which the oxide layer is built. It showed that the obtained oxide layer contained atomically 56.8% of aluminum and 43.2% of oxygen (Figure 5).

As a result of the stoichiometry calculations, its chemical composition should include 52,92% of aluminum and 47,08% of oxygen.

### 3.2. Computer Analysis in the ImageJ Program

**Figure 6:**
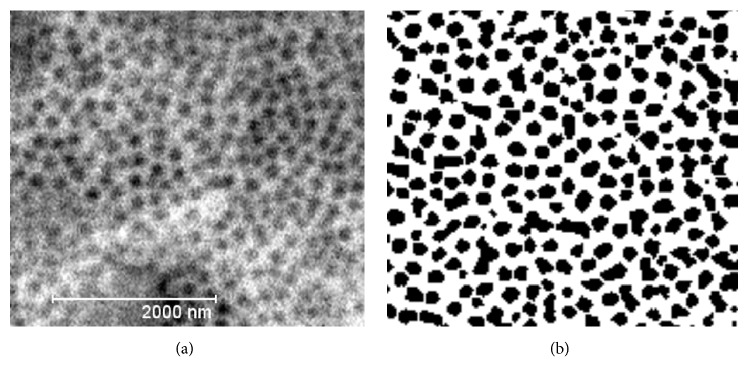
Morphology of the oxide layer produced in the electrolyte SAS: (a) SEM image and (b) binary image.

**Figure 7:**
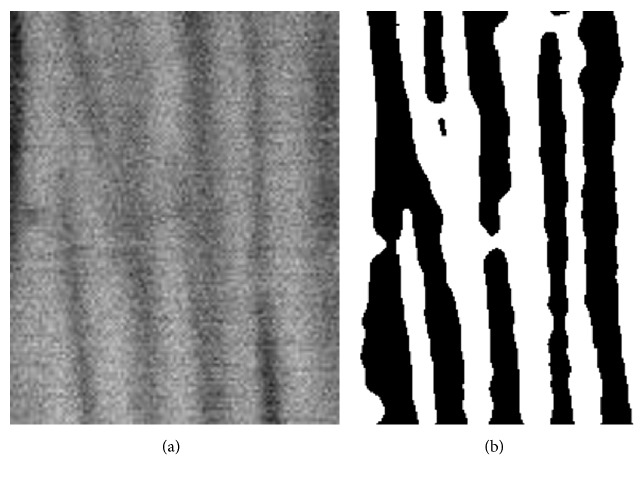
Structure of the oxide layer produced in the electrolyte SAS: (a) SEM image zoom 30,000x and (b) binary image.

To examine the Al_2_O_3_ layer's morphology and structure obtained from scanning electron microscopy images, a computer image analysis in the ImageJ program (v1.46) was used (Figures 6 and 7). The application allowed processing the actual images and their fragments and carrying out an analysis of images of the morphology of the oxide layer.

## 4. Calculation

### 4.1. The Analytical Solution

Using the properties of triangles inscribed in a circle, the properties of circles, their mutual positions between one another, and the properties of flat angles, as well as taking advantage of transformations of trigonometric functions of a triangle, the analytical solution was proposed.

The analytical solution was implemented in C++ language, the outcome of which was program code.

**Figure 8:**
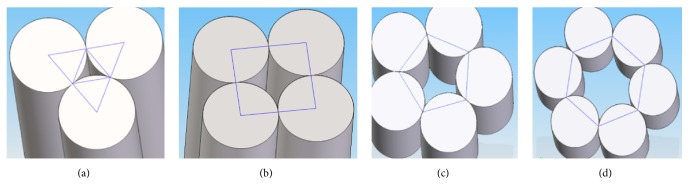
The modeled pores in the shape of (a) triangle, (b) rhombus, (c) pentagon, and (d) hexagon.

According to Skoneczny's theory [17], the possible fiber orientations were modeled in the Inventor program (Figures 8(a)–8(d)). Feret's diameters of nanopores were as follows: triangle-shaped, 50 nm, rhombus-shaped, 82 nm, pentagon-shaped, 132 nm, and hexagon-shaped, 180 nm.

**Figure 9:**
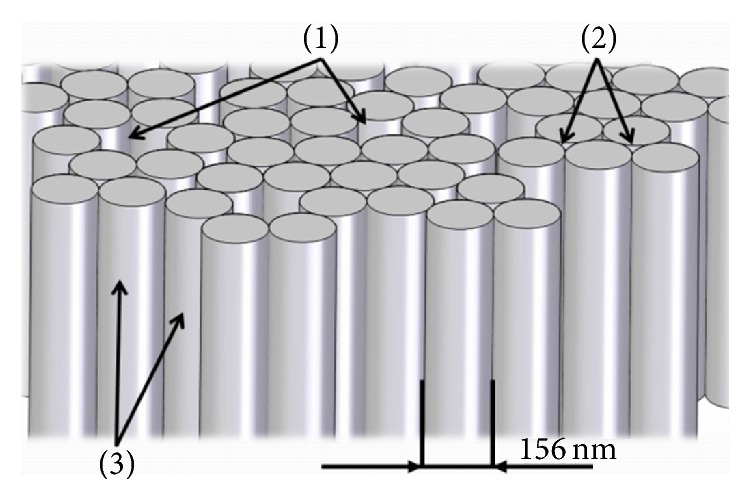
Oxide coating Al_2_O_3_ model: (1) micropores, (2) nanopores, and (3) ceramic fibers.

Based on the structure and morphology images of the Al_2_O_3_ layer, a vision of the appearance and structure of the oxide coating was obtained. A columnar model of the coating was created using CAD (Figure 9) [8].

### 4.2. The Algorithm and Functioning of the SFO 2012 Computer Program

The program was written according to the procedural algorithm:

1. Objective: to simulate the morphology of a coating and to optimize the surface topography at its design stage
2. Data: types of fiber orientations and computer analysis of SEM images
3. Problem: a program building the structure of an oxide coating
4. Problem analysis: programming fiber groups and their orientations, a mathematical study, and implementation of data, objects, and classes
5. Outcome: an application simulating the fiber orientation in a coating

**Figure 10:**
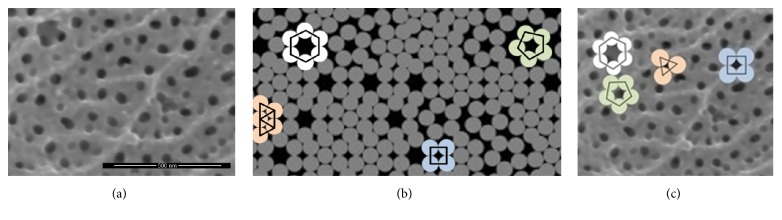
Regular groups of fibers: (a) SEM photos of AOC, (b) a window of the SFO program simulation of fiber orientation, and (c) SEM photos of AOC with markers of fiber orientation.

The operation of the program begins with the drawing of fiber groups from point 0.0 located in the top left-hand corner of the result window. The drawing area is 1000 × 500 px. The program performs a random distribution of the groups using mathematical prediction of events by means of the Monte Carlo method. It draws for one of the nanopore groups in the shape of a triangle, a pentagon, or a hexagon. A group of quadrangle nanopores is created from a combination of pentagon and hexagon groups. Image analysis showed 705 complete fiber objects that form the Al_2_O_3_ coating on the working screen of the program. The result of the operation of the application is the proposed image of the oxide coating morphology (Figures 10(a)–10(c)).

In Table 2, the comparison of nanopores size measurements obtained from SFO and microscope was shown. Measurements were made for sample B. The accuracy of the calculation in computer program results from statistical deviations of the sample and does not exceed several percent.

The surface measurements of nanopores were made using computer image analysis in ImageJ program. The application allows binarization, grouping, and calculating surface area of objects of analyzed images. Measurements are shown for exemplary sample B. The size and shape of pores depend on the substrate metal structure, the type of electrolyte, and the conditions in which the anodizing process is conducted. Nanopores, which are the result of aluminum oxide fibers contacting one another, as well as of their arrangement and growth, are formed in a perfect columnar structure without energy interference. The cross section of a pore has in that case the shape of an equilateral triangle whose sides are covered with an arc formed from a segment of a fiber. The structures of fibers creating macropores were also modeled. The macropores were formed as a result of transformation of the energy interference in the substrate structure into an oxide coating, and they took the shape of a rhombus, a quadrangle, a pentagon, or a hexagon. The appearance of a morphology obtained from a combination of various nanopores and micropores was proposed (Figure 10(b)).

## 5. Conclusions

The geometric structure of the surface is one of the most important properties of the Al_2_O_3_ coating which affects, to a large degree, its tribological characteristics in nonlubricated sliding systems.

The proposed computer model of the oxide coating's morphology built in the SFO 2012 program is an attempt to reconstruct the real system in a computer program in order to better understand and illustrate possible solutions to the investigated problem. The simulation uses a mathematical model written in the form of a computer program. The model is a simplified version of the actual system and illustrates the ongoing processes in an approximate way. The conceptual model consists of a number of assumptions that reduce the examined problem and the real area of analysis to their simplified counterparts that are acceptable in the context of the purpose of the modeling and simulation.

## Figures and Tables

**Table 1:** Parameters controlled by hard anodizing process.

Parameters marking	Current density [A/m^2^]	Electrolyte temperature [K]	Oxidation time [min]
A	200	293	40
B	300	303	60
C	400	313	80

**Table 2:** The surface measurements of nanopores.

Measured from	The shape of nanopores			
	Triangle [nm^2^]	Rhombus [nm^2^]	Pentagon [nm^2^]	Hexagon [nm^2^]
SFO program	981	5223	13227	25000
SEM image	1003	5169	13656	26696
